# Medicinal Plants for Acne Vulgaris: An Evidence‐Based Review of Treatments Promoted by Social Media

**DOI:** 10.1111/jocd.70628

**Published:** 2025-12-24

**Authors:** Mohammad Mahdi Parvizi, Nasrin Saki, Zahra Rostami Ghotbabadi, Mohammad Kamali, Nastaran Salmanpour, Mohammadreza Namazi, Mehdi Pasalar

**Affiliations:** ^1^ Molecular Dermatology Research Center Shiraz University of Medical Sciences Shiraz Iran; ^2^ Research Center for Traditional Medicine and History of Medicine Shiraz University of Medical Sciences Shiraz Iran; ^3^ Persian Medicine Network (PMN), Universal Scientific Education and Research Network (USERN) Tehran Iran; ^4^ Smart University of Medical Sciences Tehran Iran; ^5^ Department of Dermatology, School of Medicine Shiraz University of Medical Sciences Shiraz Iran; ^6^ Student Research Committee Shiraz University of Medical Sciences Shiraz Iran; ^7^ School of Medicine Shiraz University of Medical Sciences Shiraz Iran

**Keywords:** acne vulgaris, cyberspace, evidence‐based medicine, herbal medicine, Persian medicine

## Abstract

**Background:**

Acne vulgaris refers to a chronic inflammatory state of the pilosebaceous follicles that affects the majority of adolescents. Treatments encompass topical agents and systemic therapies. Nowadays, we encounter a growing tendency to use herbal remedies, which raises concerns about misinformation disseminated by digital platforms.

**Aim:**

This narrative review investigated herbal acne treatments recommended by virtual platforms (WhatsApp, Instagram, and Telegram), and gleaned medicinal plants from authoritative references.

**Methods:**

A literature search across international and Persian databases, including PubMed and Scopus, was implemented employing keywords and Boolean operators. Data synthesis highlighted evidence gaps and inconsistencies.

**Results:**

The review found different medicinal plants holding potential acne treatment properties, including tea tree oil, lavender, licorice, turmeric, and heartsease among others. Some demonstrated antibacterial, anti‐inflammatory, or sebum‐reducing effects in vitro or clinically. Others lacked strong evidence or clinical validation. Furthermore, combinations like tea tree and lavender oils showed lesion‐decreasing effects. However, gaps remain in clinical research for a number of plants traditionally claimed to treat acne vulgaris.

**Conclusion:**

While digital platforms play an important role in spreading health‐related information, they have promoted several remedies for acne vulgaris that have not been scientifically verified. Hence, cyberspace is not currently deemed a dependable source of treatment information for nonmedical experts.

## 1 Introduction

**FIGURE 1:**
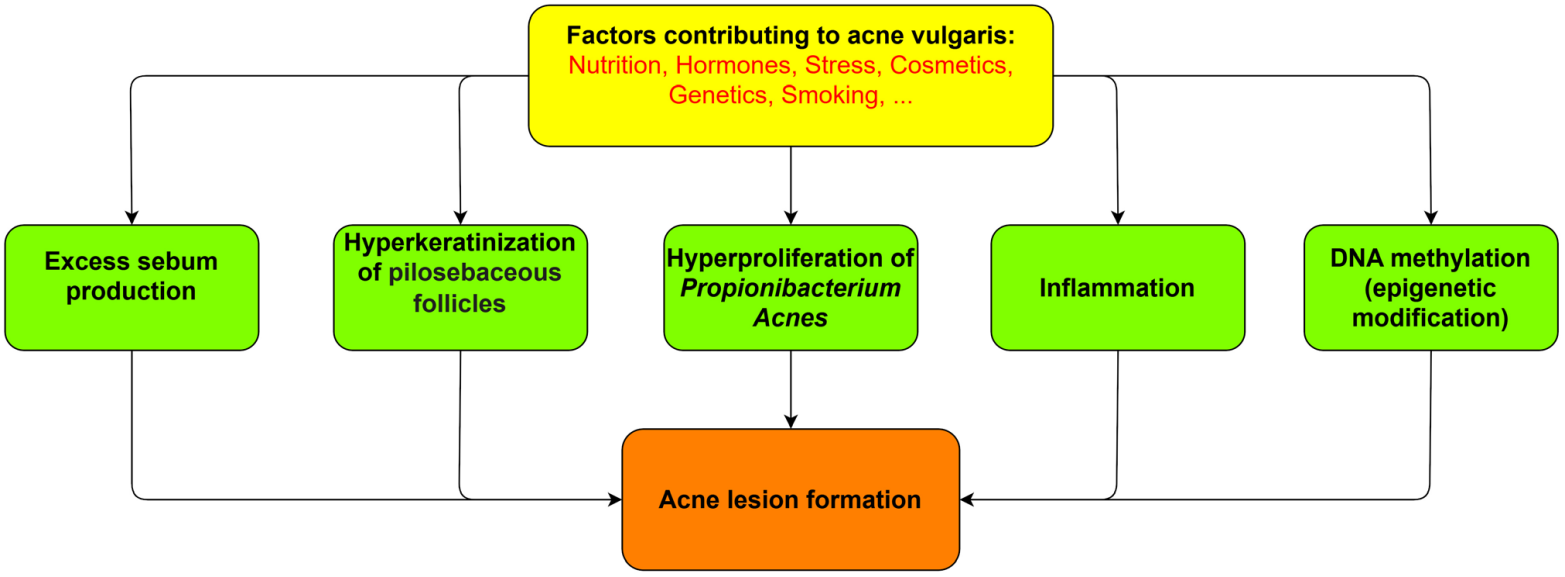
The pathogenesis underlying acne vulgaris development.

Acne vulgaris is one of the highly prevalent chronic skin disorders globally, which afflicts approximately 85% of teenagers and can also affect adults [1, 2]. This intrusive disorder impacts almost 95%–100% of adolescent boys and 83%–85% of girls and tends to affect the face and chest [3, 4]. Acne vulgaris causes chronic inflammation of sebaceous glands and leads to several manifestations, including open and closed comedones (blackheads and whiteheads), papules, pustules, nodules, and cysts. Regrettably, these skin lesions can end in scarring and hyperpigmentation [2]. Factors, such as stress, smoking, and genetic predisposition, as well as diet and drugs, contribute to the disease's activity [5]. Figure 1 outlines a general layout of the mechanisms contributing to acne vulgaris development [2].

Treatment of acne primarily pursues controlling the existing lesions and encompasses topical therapies, such as retinoids, antibiotics, and salicylic acid, as well as systemic treatments like isotretinoin and hormonal agents for more severe cases [6]. Recently, there has been a growing inclination towards utilizing herbal medications for treatment of acne, including those introduced by Traditional Persian Medicine [7, 8]. Interestingly, among those affected by dermatological diseases, acne patients comprise a group with a high tendency to seek treatment in traditional medicine [9].

On the other hand, the development of digital communication has revolutionized the healthcare landscape, which may contribute to the distribution of misleading information [10, 11]. Particularly, social media have become a source of information about herbal medicine used by a plethora of individuals [12]. Correspondingly, in our everyday evidence‐based practice, we have encountered many patients pursuing treatments proposed by so‐called traditional medicine pundits who often advertise on social media and recommend self‐medication [12, 13, 14].

This study evaluates the scientific evidence underlying the use of medicinal plants for acne treatment, which were suggested on social media platforms, specifically Instagram, WhatsApp, and Telegram. These platforms are the most prevalent and popular ones among the Iranian population. Furthermore, we intend to alleviate the spread of misinformation and promote accurate knowledge regarding the efficacy of home remedies and herbal treatments for acne.

## 2 Method

### 2.1 Study Design

This narrative review aimed to examine the scientific evidence supporting herbal medicines which are claimed in virtual spaces for the treatment of acne.

### 2.2 Data Collection From Social Media

We searched three popular social media platforms—Instagram, WhatsApp, and Telegram—to explore the online promotion of plant‐based treatments for acne vulgaris during 2022. Instead of focusing on counting posts on each platform, we intended to capture every unique treatment claim we encountered during the study period.

Owing to the constant changes of the content on these platforms, we documented posts and messages at the moment we found them, and then compiled them into a single list. Posts that were duplicated or unrelated were removed. The remaining items were categorized based on the following:

1. The plant's name.
2. The methods of using the product or herbal remedy.
3. The benefits and results claimed or other recommendations mentioned on the post.

Then, we compared these claims with the available scientific evidence to assess their validity.

### 2.3 Identification of Medicinal Plants

For each identified medicinal plant, both the scientific and common names were extracted from authoritative sources, including the Physicians' Desk Reference (PDR) and recognized reference books on medicinal plants.

### 2.4 Literature Search

Subsequently, we accessed multiple academic databases, including Google Scholar, Embase, Web of Science, Scopus, and PubMed, as well as Persian‐language databases such as Scientific Information Database (SID) and Magiran.

#### 2.4.1 Keywords and Boolean Operators

Basil oil, basil, *Ocimum basilicum* , copaiba oil, copaiba, green tea, *Rosa damascena*, rose flower, damask rose, seaweed, tea tree oil, *melaleuca alternifolia*, fenugreek, *Trigonella foenum‐graecum* , chamomile, *Matricaria chamomilla* , licorice, *Glycyrrhiza glabra* , turmeric, *Curcuma longa* , *Viola tricolor* , heartsease, *Lavandula angustifolia* , lavender, *Thymus vulgaris* , thyme, cedar, ziziphus spina‐christi, *Aloe vera* , *Senna italica* , cinnamon, eucalyptus, soapwort, *Saponaria officinalis* , coriander, *coriandrum sativum* , asparagus, *Asparagus officinalis* , acne vulgaris, acne, *Propionibacterium acnes* , *Cutibacterium acnes*.

In order to refine and broaden the search to include relevant variations and synonyms, Boolean operators (“AND” and “OR”) were also employed. Additionally, no time restrictions were applied to the search.

### 2.5 Screening and Selection Criteria

Abstracts of all identified articles were reviewed, and irrelevant or duplicate articles were excluded from further consideration. Suitable articles were included based on the predefined inclusion and exclusion criteria.

#### 2.5.1 Inclusion Criteria

- Studies investigating the effect of the specified medicinal plants on patients suffering from acne or acne‐causing bacteria.
- Research conducted in Iran or internationally.

#### 2.5.2 Exclusion Criteria

- Nonavailability of necessary results in the reports.
- Presence of similar results in other articles.
- Nonavailability of the full text of the article.

### 2.6 Data Analysis

The pertinent data were extracted. If relevant, studies were assessed from the aspects of major attributes, such as study design, sample size, blinding, and controls for confounding variables. Inconsistencies in findings across studies, if present, were noted with the emphasis on high‐quality studies and consensus guidelines from authoritative bodies. We noted the absence of scientific evidence for any plant for which no pertinent literature was found. Ultimately, a narrative synthesis approach was employed to summarize the findings.

## 3 Result

**FIGURE 2:**
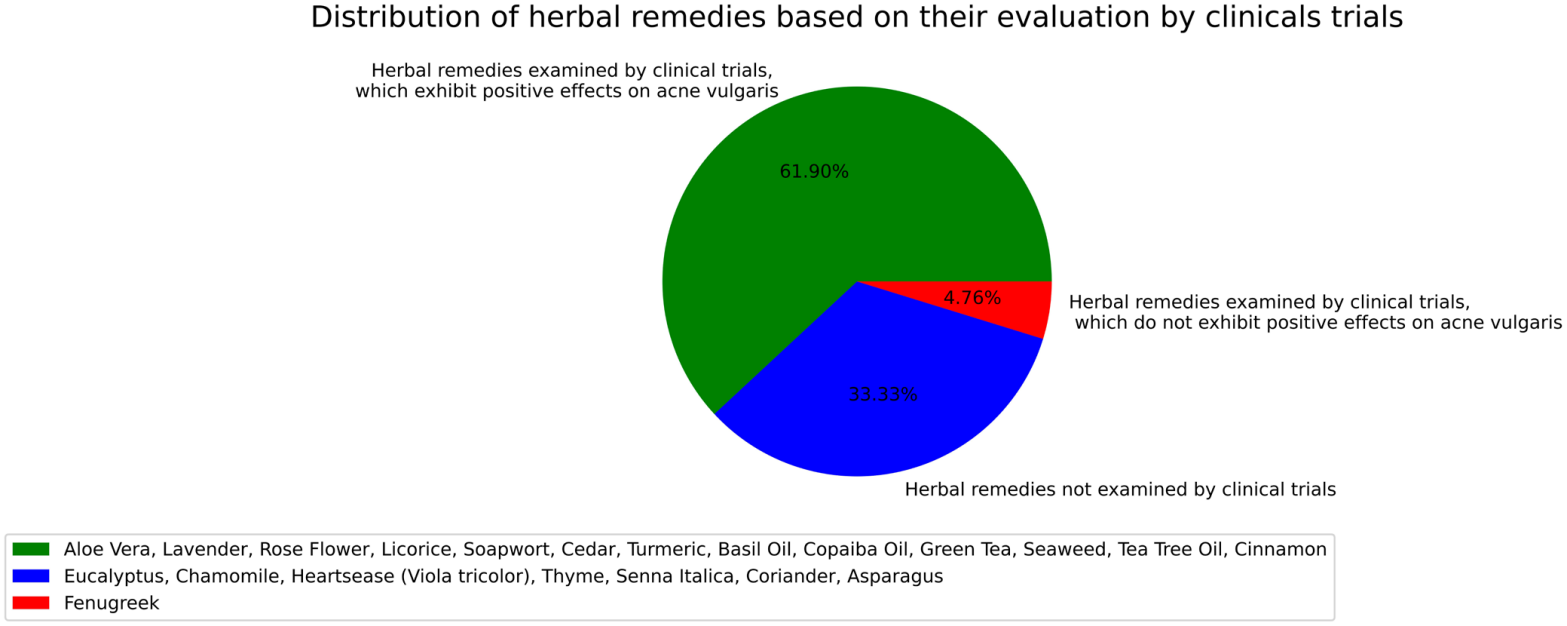
Proportions of herbal remedies by their evaluation through clinical trials.

We have totally encountered 21 herbal remedies suggested on the digital platforms, particularly by the so‐called traditional medicine experts. Fortunately, 13 held scientific approval for treating acne vulgaris with clinical trials, either when used alone or in combination with other treatments. However, 8 remedies lacked clinical trial validation despite preliminary evidence. Eventually, one remedy was not supported by any scientific studies relevant to acne treatment. Table 1 and Figure 2 give a general perspective on the findings.

**TABLE 1:** Herbal remedies, if they were examined in clinical trials, and evidence suggesting them beneficial to treat acne vulgaris.

No.	Herbal medication	Evaluated by clinical trial	Evidence relating to acne treatment
1	Fenugreek	Yes (negative results)	
2	*Aloe vera*	Yes (in combination)	*Aloe vera* gel enhances the anti‐acne effects of *Ocimum gratissimum* oil
3	Lavender	Yes (in combination)	Antibacterial effects
4	Rose Flower	Yes (in combination)	Antibacterial against *P. acnes*
5	Licorice	Yes (in combination)	Decreases testosterone level, *P. acnes* population, and sebum synthesis
6	Soapwort	Yes (in combination)	Anti‐sebum, anti‐inflammatory and antimicrobial effects (against *P. acnes* )
7	Cedar	Yes (in combination with clindamycin)	Antibacterial effects
8	Turmeric	Yes (as part of face wash)	Curcumin inhibits *P. acnes* , antioxidant and anti‐inflammatory effects
9	Basil Oil	Yes	Antibacterial against *P. acnes*
10	Copaiba Oil	Yes	Anti‐inflammatory and antimicrobial effects
11	Green Tea	Yes	Anti‐inflammatory, antioxidant, and antimicrobial effects
12	Seaweed	Yes	Decreases sebum production and *P. acnes*
13	Tea Tree Oil	Yes	Strong *P. acnes* inhibition, anti‐inflammatory effects
14	Cinnamon	Yes	Antibacterial against *P. acnes* and *S. epidermidis*
15	Eucalyptus	No	Antimicrobial activity against *P. acnes*
16	Chamomile	No	Antibacterial and anti‐inflammatory effects of methanolic extract
17	Heartsease ( *Viola tricolor* )	No	Anti‐inflammatory and antimicrobial effects
18	Thyme	No	Antibacterial effects
19	*Senna Italica*	No	Anti‐inflammatory effect
20	Coriander	No	Silver nanoparticles from coriander leaf extract show strong anti‐ *P. acnes* effect in vitro
21	Asparagus	No	

## 4 Discussion

This review highlights the growing interest in herbal remedies for acne vulgaris that are promoted on virtual platforms. Their popularity notwithstanding, our findings unveiled a remarkable disparity between the claims and the scientific evidence justifying these treatments.

### 4.1 Basil Oil

Basil oil is extracted from species like *Ocimum sanctum* , *Ocimum basilicum* , and *Ocimum gratissimum* , and has traditionally been utilized for several medical applications, such as treating insect bite [15, 16]. The oils from *O. basilicum* and *O. sanctum* possess antibacterial properties against *Propionibacterium acnes* ( *P. acnes* ), with *O. basilicum* oil exerting a stronger effect [16]. A clinical trial by Orafidiya et al. (*N* = 126) demonstrated that topical *O. gratissimum* oil reduced acne lesions more effectively and rapidly than benzoyl peroxide, with better tolerability [17].

### 4.2 Copaiba Oil

Copaiba oil, yielded from the Copaifera family, has been recognized for its anti‐inflammatory and antimicrobial properties [18]. A clinical trial by Da Silva et al. showed that a topical gel containing 1% copaiba oil significantly decreased the area of skin involved by acne lesions. These findings suggested the potential of copaiba oil for treating mild acne [19].

### 4.3 Green Tea

Green tea is known for its anti‐inflammatory, antioxidant, and antimicrobial effects ascribed to its polyphenolic compounds, particularly catechins [20]. A systematic review and meta‐analysis by Kim et al. showed that topical application of green tea extract can diminish the count of acne inflammatory and noninflammatory lesions without developing considerable adverse effects. This beneficial property was attributed to anti‐inflammatory, antioxidant, and antimicrobial effects [21]. One study demonstrated that a 3% topical emulsion of green tea lowered sebum production in healthy individuals over 60 days [22].

### 4.4 Rose Flower

Damask rose (also known as *Rosa damascena*) is a medicinal plant with a pleasant fragrance which has been used in cosmetics [23]. Its extract exhibits anti‐inflammatory and antibacterial properties against various pathogens, including *P. acnes, Staphylococcus aureus*, and *S. epidermidis* [24]. This finding suggests potential efficacy against acne vulgaris, as *P. acnes* is a key etiological factor (Figure 1). A clinical trial demonstrated that treatment of acne patients with a topical combination of retinol, rose extract, and hexamidine diisethionate resulted in a significant amelioration in terms of acne lesions counts and acne grade [25]. Nevertheless, it was not clear whether the rose extract was yielded from damask rose.

### 4.5 Seaweed

Seaweed‐derived oligosaccharides (particularly from brown seaweed ( *Laminaria digitata* )) have been found effective in mitigating acne vulgaris in clinical trials. The seaweed oligosaccharide–zinc complex (SOZC) can exert improving effects on acne via lowering production of sebum and decreasing abundance of *P. acnes* [26].

### 4.6 Tea Tree Oil

Tea tree oil, yielded from *Melaleuca alternifolia*, has been exploited for its antimicrobial and anti‐inflammatory properties [27]. A double‐blind clinical trial by Enshaieh and colleagues showed that a topical gel containing 5% tea tree oil significantly reduced acne lesions and severity [28]. The active compounds of tea tree oil, including terpinen‐4‐ol, α‐terpinolene, α‐terpinene, and α‐terpineol, possess a robust inhibitory effect on *P. acnes* [29].

### 4.7 Fenugreek

Utilization of fenugreek ( *Trigonella foenum‐graecum* ) for various ailments notwithstanding [30], a study found insufficient evidence to support its effectiveness in treating acne. Participants took either fenugreek extract or azithromycin. The results showed a decrease in acne severity; however, no significant difference in the change of mean total lesion count was observed across both groups. Thus, the study found no robust evidence affirming the fenugreek's efficacy in the treatment of acne vulgaris [31].

### 4.8 Chamomile

Chamomile ( *Matricaria chamomilla* ) has demonstrated antibacterial and antifungal effects [32]. Research indicated that its methanolic extract inhibited the growth of *P. acnes* with minimum inhibition concentration (MIC) comparable to isotretinoin. The extract showed potent antibacterial and anti‐inflammatory effects, which rendered it a promising option for acne treatment [33]. Nonetheless, we found no randomized clinical trial confirming the effectiveness of chamomile in mitigating acne vulgaris lesions.

### 4.9 Licorice

Licorice ( *Glycyrrhiza glabra* ) possesses anti‐inflammatory properties. Licorice extract is effective against *P. acnes* and can decrease sebum synthesis, thereby contributing to acne improvement [34]. Moreover, licorice extracts diminish the activity of the 11β‐hydroxysteroid dehydrogenase (11β‐HSD) enzyme converting androgenic steroids into testosterone; therefore, the extract ultimately reduces serum testosterone content [35], which plays a role in developing acne vulgaris [2]. A clinical trial by Iraji and colleagues showed an herbal cream containing *Aloe vera* leaves, pomegranate peel, licorice root, and zataria leaves extract significantly reduced acne severity index and total lesion counts after 2 months of utilization [36].

### 4.10 Turmeric

Turmeric ( *Curcuma longa* ), primarily popular for its active component (curcumin), holds antioxidant and anti‐inflammatory properties [37]. Research has unveiled that curcumin effectively inhibits the growth of *P. acnes* , which supports its use as a topical treatment for acne [38]. A clinical trial by Rajaiah Yogesh et al. assessed the efficacy and safety of Purifying Neem Face Wash (PNFW), containing turmeric, to avert and improve acne vulgaris. The study showed that PNFW can benefit acne vulgaris treatment and prevention without causing any side effects [39].

### 4.11 Heartsease

*Viola tricolor* was among the medicinal plants traditionally utilized for acne treatment [40]. Correspondingly, studies have demonstrated that this plant has anti‐inflammatory and antimicrobial properties, which may justify its use in the treatment of acne vulgaris [41]. In addition, this plant contains salicylic acid, which is used for treating acne [42]. Nonetheless, no clinical trial has specifically evaluated the implications of *Viola tricolor* for acne.

### 4.12 Lavender

*Lavandula angustifolia* is a pleasantly fragrant shrub whose flowers are utilized to yield lavender oil via distillation. Despite the antibacterial activity of lavender oil, commercially available lavender oil did not exert an effective bactericidal effect on *P. acnes* . Seemingly, the proportion of linalool, one of the oil's constituents, determines the bactericidal effectiveness. Accordingly, this oil is recommended for superficial infectious afflictions, rather than deep ones. A study has shown the considerable reduction in the population of *P. acnes* and acne inflammatory lesions following a four‐week application of a mixture of tea tree and lavender oils. These findings suggest that lavender oil could be an alternative treatment for acne in individuals who are reluctant to use antibiotics [43].

### 4.13 Thyme

Thyme ( *Thymus vulgaris* ) has been recognized for its medicinal and culinary applications. Its active compounds, including saponins, flavonoids, and phenolics, contribute to its therapeutic effects [44]. A study accomplished by Taleb and colleagues in 2018 evaluated the antibacterial effects of thyme oil against *Propionibacterium acnes* and *Staphylococcus epidermidis* . The results indicated a minimum inhibitory concentration (MIC) of 0.7 mg/mL for both bacteria, with minimum bactericidal concentrations (MBC) of 1.4 mg/mL for *P. acnes* and 2.8 mg/mL for *S. epidermidis* . Although oregano exhibited a greater antibacterial effect, thyme's efficacy in combating acne‐causing bacteria is notable, especially in the context of rising antibiotic resistance [45]. Further research by Mohammed et al. in 2020 showed that alcoholic extracts of thyme had a stronger antimicrobial effect compared to its oil, suggesting that thyme extracts could be beneficial in acne treatment [46].

### 4.14 Cedar

Cedar (*Ziziphus spina‐christi*) is a thorny tree containing saponin compounds and has exhibited antibacterial effects [47, 48]. A double‐blind trial carried out by Shakiba et al. in 2019 included 68 patients with mild to moderate acne. The results revealed that the group receiving a topical cedar solution alongside clindamycin showed a significant diminution in acne severity and lesion count in comparison with the control group solely using clindamycin. This suggests a potential role for cedar in acne management, despite the need for further studies with larger cohorts to corroborate these findings [48].

### 4.15 *Aloe vera*

*Aloe vera* , a succulent plant, holds soothing (itch‐ and pain‐relieving effects) and anti‐inflammatory properties [49]. A study by Mazzarello et al. in 2018 compared treatment of mild to moderate acne with a cream containing propolis, tea tree oil, and *Aloe vera* and with erythromycin cream. The combination cream showed superiority in terms of reducing redness, count of lesions, and overall acne severity [50]. Furthermore, a study by Orafidiya et al. in 2004 demonstrated that *Aloe vera* gel augmented the anti‐acne properties of *Ocimum gratissimum* oil, which surpassed clindamycin in efficacy [51]. These studies indicate that *Aloe vera* could be a valuable component in acne treatment regimens.

### 4.16 *Senna italica*

*Senna italica* has been claimed by the traditional medicine fonds as a treatment option for acne vulgaris. This medicinal plant has purgative, anti‐inflammatory, and antioxidant effects [52]. However, to our best knowledge, there is no study substantiating the application of *Senna italica* against acne vulgaris. Moreover, no clinical trial examining this application was found.

### 4.17 Cinnamon

Cinnamon bark extract has demonstrated antibacterial properties against *P. acnes* and *S. epidermidis* . A study by Julianti et al. in 2016 found that cinnamon extract, both alone and in combination with honey, exhibited significant antibacterial activity against the aforementioned bacteria [53]. Another clinical trial by Ghovvati et al. in 2018 showed that a cinnamon gel applied to patients with mild to moderate acne involving the face resulted in a notable reduction in both inflammatory and noninflammatory lesions over 8 weeks. Despite the reports of mild burning sensations and transient redness, the overall findings suggest that cinnamon is effective in treating facial acne [54].

### 4.18 Eucalyptus

Volatile oils extracted from eucalyptus were investigated by Athikomkulchai et al. in 2008. Their study focused on the volatile oils of eucalyptus leaves, which showed significant antimicrobial activity against *P. acnes* , with MIC and MBC values of 9.38 mg/mL. The combination of eucalyptus and guava oils was formulated into creams that maintained stability and demonstrated effective antimicrobial properties. Compared to a 5% benzoyl peroxide gel, eucalyptus oil exhibited considerable antibacterial activity (against *P. acnes* ), suggesting its feasibility as an alternative treatment option [55].

### 4.19 Soapwort

Soapwort ( *Saponaria officinalis* ) is a flowering plant carrying compounds such as saponins and flavonoids. This herb exerts anti‐inflammatory, antimicrobial, and antifungal effects, rendering it a remedy hailed by traditional medicine [56]. A clinical trial by Said et al. has proven soapwort's anti‐sebum synthesis, anti‐inflammatory, and antimicrobial effects (against *P. acnes* ) in vitro. Additionally, during the study, an herbal cream was formulated from *Saponaria officinalis, Inula helenium*, and *Solanum nigrum* , which resulted in a significant decline in the numbers of acne inflammatory and noninflammatory lesions [57].

### 4.20 Coriander

Coriander ( *Coriandrum sativum* ) is a medicinal herb offering several merits, including antibacterial properties. A study by Sathishkumar and colleagues in 2016 investigated the anti‐acne properties of silver nanoparticles derived from coriander leaf extract. Utilizing various analytical techniques, the study found a MIC of 3.1 μg/mL against *P. acnes* , indicating a significant antimicrobial effect. These results suggest that coriander may be effective in treating acne, but additional research with larger cohorts is necessary to validate these findings [58].

### 4.21 Asparagus

Historically, asparagus ( *Asparagus officinalis* ) has been exploited for medicinal and culinary purposes, considering its bioactive constituents. Studies have provided insights into the valuable effects of asparagus, such as immunomodulation and anti‐oxidation [59]. However, although there are several recommendations on cyberspace for utilizing asparagus for treatment of acne vulgaris, no clinical trial has been implemented to confirm its presumptive benefits for acne.

### 4.22 Adverse Effects

There is a dearth of studies examining the adverse effects of these medicinal plants when specifically exploited to treat acne vulgaris. Also, the frequency and likelihood of the adverse effects have not been thoroughly investigated yet if these medications are used for acne vulgaris treatment.

The majority of adverse effects comprise local skin irritation. *Ocimum* oil has been revealed to possess skin‐irritating effects when used at concentrations of 5% v/v, except when dissolved in a petrolatum base. However, concentrations of 2% v/v showed no skin irritation, regardless of the base utilized [17]. Similarly, topical copaiba oil can induce erythema and pruritus [60].

*Aloe vera* is generally tolerated well, despite occasional reports about hypersensitivity and eczematous dermatitis after utilizing *Aloe vera* , either orally or topically [61]. Moreover, lavender can induce allergic contact dermatitis. This occurrence is uncommon yet important [62].

Notwithstanding extensive use of rose flower, a case of allergic contact dermatitis triggered by *Rosa mosqueta* oil has been reported, which warrants caution in leveraging rose flower extracts [63]. Additionally, topical licorice‐containing products utilization has been associated with occasional occurrence of contact dermatitis [64]. Topical application of turmeric, cinnamon, or tea tree oil can lead to allergic contact dermatitis [65, 66, 67]. It should not be overlooked that allergic contact stomatitis can also involve the patient if cinnamon oil is administered orally [66]. Topical seaweed can provoke an irritant contact dermatitis [68]. This can rarely cause anaphylactic reaction [69].

Epigallocatechin gallate, the main active constituent of green tea extract, has been reported to cause skin irritation in rats and guinea pigs when used topically [70]. In addition, skin erythema occurred in a breast‐cancer patient who had undergone radiotherapy, following topical application of epigallocatechin gallate intended to mitigate the radiotherapy‐induced skin injury [71].

On the other hand, endocrinologic disturbances were also reported. A case report demonstrated gynecomastia involving a number of prepubertal boys who topically used a product consisting of oils of lavender and tea tree. Strikingly, the article inferred a causal association between frequent use of the product and prepubertal breast growth [72]. Furthermore, using lavender‐including fragrance has been associated with prepubertal gynecomastia in boys, as well as premature thelarche in girls, in a few cases. These phenomena were attributed to the estrogenic and antiandrogenic effects lavender's essential oil exerted. However, breast enlargement regressed following cessation of utilization of the suspicious products [73]. Notably, pseudoaldosteronism may occur amid oral consumption of licorice‐containing drugs, brought about by glycyrrhizin of licorice. However, considering the risk factors, the occurrence of pseudoaldosteronism seems unlikely with topical application of licorice‐containing drugs [74].

Importantly, basil oils can exert hypoglycemic [75], hypotensive [76], and blood‐thinning effects [77] when administered orally. Although it is applied topically for the treatment of acne vulgaris, the possibility of these effects should be taken into account.

Although rarely reported, orally administered turmeric seemingly holds the potential to cause liver injury [78].

Basil oil, copaiba oil, green tea, seaweed, tea tree oil, and cinnamon have shown beneficial effects of their derivatives, such as extracts or essential oils, in clinical trials. Similarly, clinical trials have demonstrated that topical formulations containing *Aloe vera* , lavender, rose flower, licorice, soapwort, cedar, or turmeric, when combined with other therapeutic agents, mitigate acne vulgaris. In contrast, a clinical trial found no robust evidence supporting the use of fenugreek extract compared to oral azithromycin for treating acne vulgaris. Nonetheless, patients may prefer using herbal remedies over oral chemical agents, such as azithromycin—the commonly recommended treatment for acne vulgaris—due to concerns about the potential adverse effects of azithromycin, which include QT prolongation, hepatotoxicity, *Clostridioides difficile* infection, nausea, dyspepsia, flatulence, diarrhea, constipation, cholestatic jaundice, and, rarely, erythema multiforme, toxic epidermal necrolysis, and Steven‐Johnson syndrome [79, 80].

On the other side, six remedies, such as eucalyptus, chamomile, *viola tricolor* , thyme, *senna italica* , and coriander, lacked direct clinical trial evidence for acne despite exhibiting beneficial antibacterial, anti‐inflammatory, or antioxidant effects in vitro or in combination therapies. These findings suggest potential yet inconclusive merits due to lack of clinical research. Ultimately, the use of asparagus for treating acne was not supported by any clinical trials or by indirect evidence linking asparagus to the pathogenesis of acne.

Importantly, the identification of remedies, such as asparagus, was worrisome. In fact, this was recommended online without any supporting scientific evidence for acne treatment. This finding underscores the risk of misinformation, which may mislead users and delay effective treatment or even cause harm.

Fortunately, the therapeutic properties of the scientifically supported herbs in this review align with the multifactorial pathogenesis of acne vulgaris.

The main limitation we faced in this study was the continual changes of social media content. Correspondingly, while we were collecting data, some posts and messages were edited, deleted, or made unavailable; thus, it was impossible to present the exact number of the posts on each platform. This issue also contributed to a hindrance to tracking the origin of the posts. Accordingly, we focused on documenting unique treatment claims rather than comparing platforms numerically. Future research leveraging real‐time capturing tools or long‐term monitoring would provide more stable and measurable data. Moreover, this study solely involved the Persian‐language social media content and particularly focused on the claims related to Persian traditional medicine. Consequently, treatment approaches suggested by other traditional and complementary systems, including Ayurveda, Indian medicine, or homeopathy, despite utilizing medicinal plants, were not included in our study. Importantly, these medical systems are not popular among the Iranian population. Further studies should examine and incorporate various traditional, complementary, and folk medicines in order to offer a more comprehensive perspective.

## 5 Conclusion

Although several herbal remedies show promise and can complement conventional acne management, some fake practitioners create and spread deceptive social media content lacking any evidence‐based underpinnings. To minimize the potential side effects of using botanical remedies, widespread public education and awareness are essential. Individuals should be encouraged to critically evaluate treatment claims shared on social media and avoid trusting unverified cyberspace content. Also, prior to following such recommendations, they should consult the expert traditional and complementary medicine practitioners. Additionally, professionals and researchers should expand their research to include the efficacy, safety, and adverse effects of medicinal plants. By propagating scientifically validated information through digital contents, professionals may significantly influence the public perception, enabling safer medical practice.

## Author Contributions

Study concept and design: Mohammad Mahdi Parvizi, Nasrin Saki, Zahra Rostami Ghotbabadi. Acquisition of data: Mohammad Mahdi Parvizi, Nasrin Saki, Mohammad Kamali, Nastaran Salmanpour, Mehdi Pasalar. Analysis and interpretation of data: Mohammad Mahdi Parvizi, Nasrin Saki, Zahra Rostami Ghotbabadi, Mohammad Kamali, Nastaran Salmanpour, Mohammadreza Namazi, Mehdi Pasalar. Drafting of the manuscript: Mohammad Mahdi Parvizi, Nasrin Saki, Mohammad Kamali, Nastaran Salmanpour, Mohammadreza Namazi. Finalizing the manuscript: Mohammad Mahdi Parvizi, Nasrin Saki, Zahra Rostami Ghotbabadi, Mohammad Kamali, Nastaran Salmanpour, Mohammadreza Namazi, Mehdi Pasalar. Critical revision of the manuscript for important intellectual content: Mohammad Mahdi Parvizi, Mohammad Kamali, Mehdi Pasalar. Administrative, technical, and material support: Mohammad Mahdi Parvizi, Nasrin Saki. Study supervision: Mohammad Mahdi Parvizi.

## Funding

This study was funded by the Vice Chancellery of Research of Shiraz University of Medical Sciences (proposal number: 21060).

## Ethics Statement

This study was approved by the Research Ethics Committee of Shiraz University of Medical Sciences (ID: IR.SUMS.MED.REC.1399.504).

## Consent

The authors have nothing to report.

## Conflicts of Interest

The authors declare no conflicts of interest.

## Data Availability

Data sharing not applicable to this article as no datasets were generated or analyzed during the current study.
